# Nomogram based on albumin and neutrophil-to-lymphocyte ratio for predicting the prognosis of patients with oral cavity squamous cell carcinoma

**DOI:** 10.1038/s41598-018-31498-z

**Published:** 2018-08-30

**Authors:** Huang-Kai Kao, Jonas Löfstrand, Charles Yuen-Yung Loh, William Wei-Kai Lao, Jui-Shan Yi, Yu-Liang Chang, Kai-Ping Chang

**Affiliations:** 1 Department of Plastic and Reconstructive Surgery, Chang Gung Memorial Hospital, Taoyuan, Taiwan; 2 College of Medicine Chang Gung University, Taoyuan, Taiwan; 3 Department of Plastic Surgery, Sahlgrenska University Hospital, Gothenburg, Sweden; 4 Department of Otolaryngology - Head & Neck Surgery, Chang Gung Memorial Hospital, Taoyuan, Taiwan; 5 St Andrew’s Center for Plastic and Reconstructive Surgery, Broomfield Hospital, Chelmsford, Essex, UK; 6 Department of Oral & Maxillofacial Surgery, Chang Gung Memorial Hospital, Taoyuan, Taiwan

## Abstract

Increasing evidence indicates that inflammation plays a crucial role in cancer development. A novel scoring system based on albumin and the neutrophil-to-lymphocyte ratio (NLR) was developed and incorporated into a nomogram to create a more accurate prognostic tool for oral cavity squamous cell carcinoma (OSCC) patients. A retrospective review was performed on 613 consecutive patients undergoing ablative surgery for OSCC between September 2005 and December 2014. NLR and albumin were determined and used to calculate an albumin/NLR score (ANS). The nomogram was based on the ANS and several clinicopathological manifestations, and its accuracy was determined by the concordance index (c-index). A high ANS was significantly associated with aggressive tumor behaviors, such as T status, overall stage, extranodal extension, perineural invasion, tumor depth, and decreased overall survival (OS). Multivariate analysis indicated that age, overall stage, extranodal extension, and ANS were independent factors for OS. The c-index for OS prognosis was 0.750 using this nomogram compared to 0.688 using TNM staging alone. The prognostic accuracy for OS in OSCC patients can be significantly improved using a nomogram that incorporates the novel ANS and other clinicopathological variables.

## Introduction

Oral cavity cancer is one of the most common cancers worldwide, with an estimated annual incidence of approximately 300,000 cases^1^. The dominant histologic type of oral cancer is oral cavity squamous cell carcinoma (OSCC). The prognosis for overall survival (OS) and risk of recurrence depends on both tumor-specific factors such as tumor size, nodal status, distant metastasis, extranodal extension (ENE) and bone invasion as well as patient-specific factors such as age, smoking, race, comorbidities and sex^2–6^. Although the TNM classification is important for predicting clinical outcomes and to serve as a guide for ablative and reconstructive treatment, OS vary greatly, even in patients at the same disease stage^7,8^. Recent reports have suggested that patient prognosis is associated with certain molecular biomarkers involved in angiogenesis, cell mutation, proliferation and differentiation. However, expensive laboratory techniques and comprehensive tests are required. Identifying biomarkers, particularly serum biomarkers, is crucial for practical clinical application and would help clinicians adopt preventive and therapeutic strategies for OSCC patients.

Increasing evidence has indicated that cancer development and progression is determined by both tumor characteristics and systemic inflammatory responses^9–12^. In certain types of cancers, some of these inflammation factors have shown to have prognostic value, including C-reactive protein, albumin, neutrophil-to-lymphocyte ratio (NLR), and hemoglobin^13–18^. Some authors have combined biomarkers to increase prognostic value, such as the systemic inflammation score, which combines both the lymphocyte-to-macrophage ratio and albumin, and the Glasgow prognostic score, which combines C-reactive protein and albumin levels^19,20^. Few studies have been conducted on the prognostic value of the above-mentioned biomarkers in oral cancer^21,22^.

Nomograms are statistical tools that use two or more known variables to calculate an outcome/result^23^. Nomograms are common in the oncology field for calculating the prognosis of different cancers using different variable sets based on cancer type. However, nomograms for predicting OSCC outcomes are scarce. This study introduces a novel albumin/NLR score (ANS) that, when combined with other prognostic, demographic and clinicopathological factors, can create a nomogram to predict the 3- and 5-year OS for OSCC patients after surgery.

## Results

### Patient characteristics and clinicopathological data

Among the 613 patients, 556 (90.7%) were male, and 57 (9.3%) were female. The most common cancer site was the buccal mucosa (37%), followed closely by the tongue (36.9%). Of the patients, 83% were smokers, 67.4% were alcohol consumers, and 79.6% were betel nut chewers. The mean age was 53.0 ± 11.38 years, with a range from 21.9 to 88.7 years. The most common TNM stage was IV (43.7%), followed by II (23.2%). All characteristics are described in Table 1. Dichotomization of patients by albumin and NLR levels was performed using the median value as a cut-off, which was 4.5 g·l^−1^ for albumin and 2.28 for NLR.

**Table 1:** Clinical, pathological and laboratory characteristics of the patients.

Characteristics	Number	Percentage %
Sex		
Male	556	90.7
Female	57	9.3
Lesion site		
Buccal mucosa	226	36.9
Mouth floor	32	5.2
Gingiva	89	14.5
Hard palate	17	2.8
Lip	22	3.6
Tongue	227	37.0
Tumor size (T)		
T1	126	20.6
T2	200	32.6
T3	72	11.8
T4	215	35.0
Nodal metastasis		
N0	402	65.6
N1	85	13.9
N2	126	20.5
TNM staging		
I	109	17.8
II	142	23.2
III	94	15.3
IV	268	43.7
Cell differentiation		
W-D	200	32.7
M-D	343	56.1
P-D	68	11.1
Alcohol consumption		
No	200	32.6
Yes	413	67.4
Betel nut chewing		
No	125	20.4
Yes	488	79.6
Cigarette smoking		
No	104	17.0
Yes	509	83.0
Treatment		
Surgery only	287	44.8
Surgery + Radiotherapy	109	17.8
Surgery + chemoradiotherapy	217	35.4
**Characteristics**	**Number**	**Mean** ± **SD (Max, Min)**
Age (Years)	613	53.0 ± 11.38 (88.7, 21.9)
Body mass index	613	24.1 ± 4.1 (46.5, 11.9)
Albumin (gl^−1^)	613	4.4 ± 0.3 (5.6, 2.5)
NLR*	613	2.7 ± 1.78 (22.5, 0.6)

*NLR: neutrophil-to-lymphocyte ratio.

### Prognostic factors

Dichotomization of patients by albumin and NLR levels was performed using the median value as a cut-off, which was 4.5 g·l^−1^ for albumin and 2.28 for NLR. To calculate the ANS, values below the cut-off value for albumin and over the cut-off value for NLR were given 1 point each, giving each patient a score from 0–2. A high ANS was significantly associated with high overall stage, ENE, perineural invasion, and tumor depth (Table 2). Univariate analysis revealed that indicators of poor OS prognostic value were patient age of over 65 years, increased tumor stage, perineural invasion, ENE, poor cell differentiation, increased tumor depths, and less (<5 mm) surgical margins (Table 3). Multivariate analysis indicated that age, overall stage, ENE, and ANS were independent factors of OS (*p* = 0.022, *p* = 0.017, *p* < 0.0001, and *p* = 0.040, respectively) (Table 3).

**Table 2:** Associations of albumin, NLR and ANS with clinicopathological characteristics.

Characteristics	Albumin			NLR			ANS			
	≥4.5	<4.5	*P*-values	≤2.28	>2.28	*P*-values	0	1	2	*P*-values
	n = 313	n = 300		n = 307	n = 306		n = 180	n = 260	n = 173	
Age (years)^§^	50.3 ± 10.4 (84.1, 21.9)	55.7 ± 11.7 (88.7, 30.9)	<0.0001^¶^	52.8 ± 11.5 (86.8, 21.9)	53.1 ± 11.2 (88.7, 30.3)	0.8701	49.2 ± 9.5 (73.5, 21.9)	54.8 ± 12.1 (86.8, 30.5)	54.1 ± 11.1 (88.7, 30.9)	<0.0001^¶^
Body mass index^§^	24.6 ± 4.1 (39.7, 11.9)	23.8 ± 4.1 (46.5, 12.0)	0.0110^¶^	24.8 ± 4.2 (41.5, 12.0)	23.7 ± 3.9 (46.5, 11.9)	0.0026^¶^	24.9 ± 4.3 (39.7, 15.1)	24.3 ± 4.0 (41.5, 11.9)	23.3 ± 3.9 (46.5, 15.5)	0.0006^¶^
Sex										
Male	288	268	0.2535	272	284	0.0726	166	228	162	0.0797
Female	25	32		35	22		14	32	11	
pT Status										
1–2	189	136	0.0002^¶^	212	113	<0.0001^¶^	125	151	49	<0.0001^¶^
3–4	123	164		94	193		54	109	124	
pN Status										
(−)	213	189	0.1882	213	189	0.0472^¶^	125	176	101	0.0585
(+)	100	111		94	117		55	84	72	
Overall Pathological Stage										
I-II	148	103	0.0011^¶^	168	83	<0.0001^¶^	99	118	34	<0.0001^¶^
III-IV	165	197		139	223		81	142	139	
ENE										
(−)	259	235	0.1405	265	229	0.0002^¶^	155	214	125	0.0016^¶^
(+)	52	64		40	76		24	44	48	
Cell Differentiation*										
W-D + M-D	279	264	0.6574	277	266	0.1262	164	228	151	0.3691
P-D	33	35		28	40		15	31	22	
Perineural Invasion										
No	215	192	0.2191	222	185	0.0019^¶^	127	183	97	0.0032^¶^
Yes	98	108		85	121		53	77	76	
**Tumor Depth (mm)** ^**§**^	10.3 ± 9.1 (50.0, 0.0)	13.9 ± 11.9 (68.0, 0.3)	<0.0001^¶^	9.2 ± 7.9 (65.0, 0.0)	14.9 ± 12.3 (68.0, 0.0)	<0.0001^¶^	9.1 ± 7.4 (37.0, 0.0)	10.7 ± 9.8 (65.0, 0.0)	17.3 ± 13.0 (68.0, 1.0)	<0.0001^¶^

Abbreviations: ENE: extranodal extension; NLR = neutrophil-to-lymphocyte ratio; ANS = albumin/NLR score.

*W-D: well-differentiated, M-D: moderately differentiated, and P-D: poorly differentiated, squamous cell carcinoma.

^§^Mean ± SD (Maximum, Minimum).

^**¶**^Statistically significant.

**Table 3:** Univariate and multivariate analysis of OS in patients with oral cavity squamous cell carcinoma after treatment.

Variables	Univariate			Multivariate		
	Hazards Ratio	95% CI	*P*-value	Hazards Ratio	95% CI	*P*-value
Age (Years)						
≤65	Reference			Reference		
>65	1.435	1.011–2.039	0.0434^†^	1.528	1.063–2.197	0.0220^†^
Sex						
Male	Reference			Reference		
Female	1.226	0.779–1.928	0.3781	1.158	0.725–1.850	0.5381
Overall Pathological Stage						
I	Reference			Reference		
II	2.345	1.110–4.954	0.0256^†^	2.603	1.181–5.738	0.0177^†^
III	3.679	1.735–7.798	0.0007^†^	3.391	1.504–7.644	0.0032^†^
IV	7.690	3.912–15.117	<0.0001^†^	4.928	2.213–10.974	<0.0001^†^
ENE						
(−)	Reference			Reference		
(+)	3.665	2.725–4.929	<0.0001^†^	2.096	1.477–2.975	<0.0001^†^
Perineural Invasion						
No	Reference			Reference		
Yes	1.926	1.453–2.553	<0.0001^†^	1.061	0.764–1.473	0.7222
Cell Differentiation*						
W-D + M-D	Reference			Reference		
P-D	1.916	1.305–2.814	0.0009^†^	1.359	0.908–2.034	0.1363
Tumor Depth (mm)						
<10 mm	Reference			Reference		
≥10 mm	2.423	1.801–3.259	<0.0001^†^	1.119	0.773–1.619	0.5517
Surgical margin						
<5 mm	Reference			Reference		
≥5 mm	0.696	0.518–0.934	0.0158^†^	0.914	0.675–1.238	0.5622
Albumin (gl^−1^)						
≥4.5	Reference					
<4.5	2.109	1.575–2.825	<0.0001^†^			
NLR						
≤2.28	Reference					
>2.28	1.759	1.320–2.345	0.0001^†^			
ANS						
score = 0	Reference			Reference		
score = 1	1.802	1.201–2.703	0.0044^†^	1.550	1.020–2.354	0.0400^†^
score = 2	3.181	2.124–4.763	<0.0001^†^	1.962	1.283–3.001	0.0019^†^

Abbreviations: OS = overall survival; CI = confidence interval; ENE = extranodal extension; NLR = neutrophil-t-lymphocyte ratio; ANS = albumin/NLR score.

*W-D = well-differentiated, M-D = moderately differentiated, and P-D = poorly differentiated, squamous cell carcinoma.

^†^Statistically significant.

**Figure 1:**
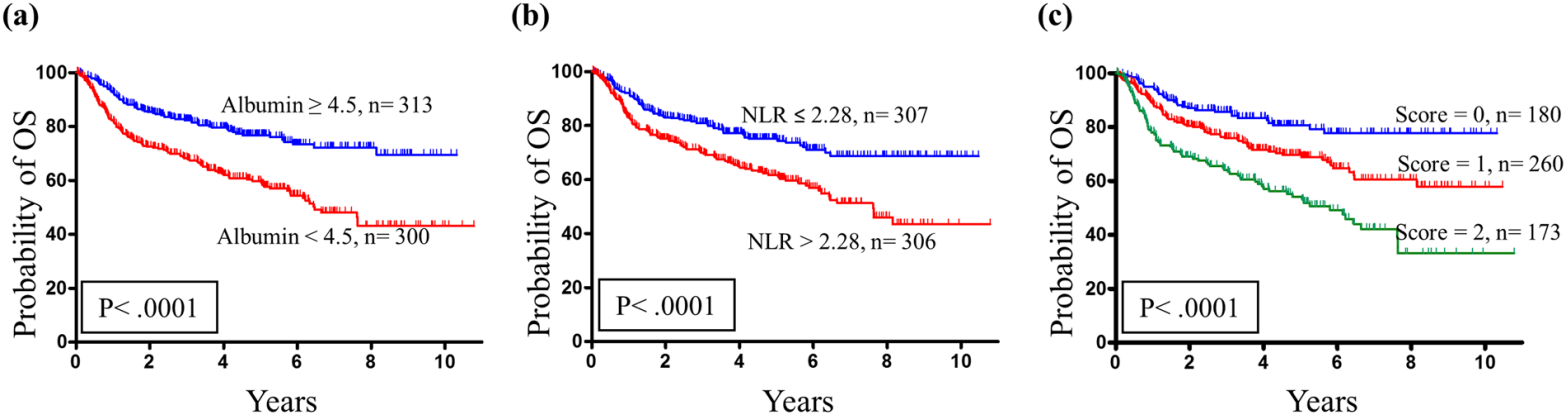
Association of albumin, NLR and ANS with the probability of overall survival (OS). (**a**) Kaplan-Meier plot for OS probability, where the 5-year OS rates for patient subgroups stratified by albumin were 77.6% *vs*. 58.0% (*p* < 0.0001). (**b**) Kaplan-Meier plot for OS probability, where the 5-year OS rates for patient subgroups stratified by NLR were 75.2% *vs*. 60.7% (*p* < 0.0001). (**c**) Kaplan-Meier plot for OS probability, where the 5-year OS rates for patient subgroups stratified by ANS score were 81.6% *vs*. 69.2% *vs*. 52.0% (*p* < 0.0001).

The OS probability analysis revealed that the five-year OS incidence for patients stratified into albumin ≥4.5 g·l^−1^ and <4.5 g·l^−1^ subgroups were 77.6% and 58.0%, respectively. These differences in OS were significant based on the log-rank test (*p* < 0.0001; Fig. 1a). The OS probability analysis revealed that the five-year OS incidence for patients stratified into NLR ≤2.28 and >2.28 subgroups were 75.2% and 60.7%, respectively. These differences in OS were significant based on the log-rank test (*p* < 0.0001; Fig. 1b). The OS probability analysis revealed that the five-year incidence of OS for patients stratified into the ANS = 0, 1 and 2 subgroups were 81.6%, 69.2% and 52.0%, respectively. The differences in OS were significant by the log-rank test (*p* < 0.0001; Fig. 1c).

### Nomogram

**Figure 2:**
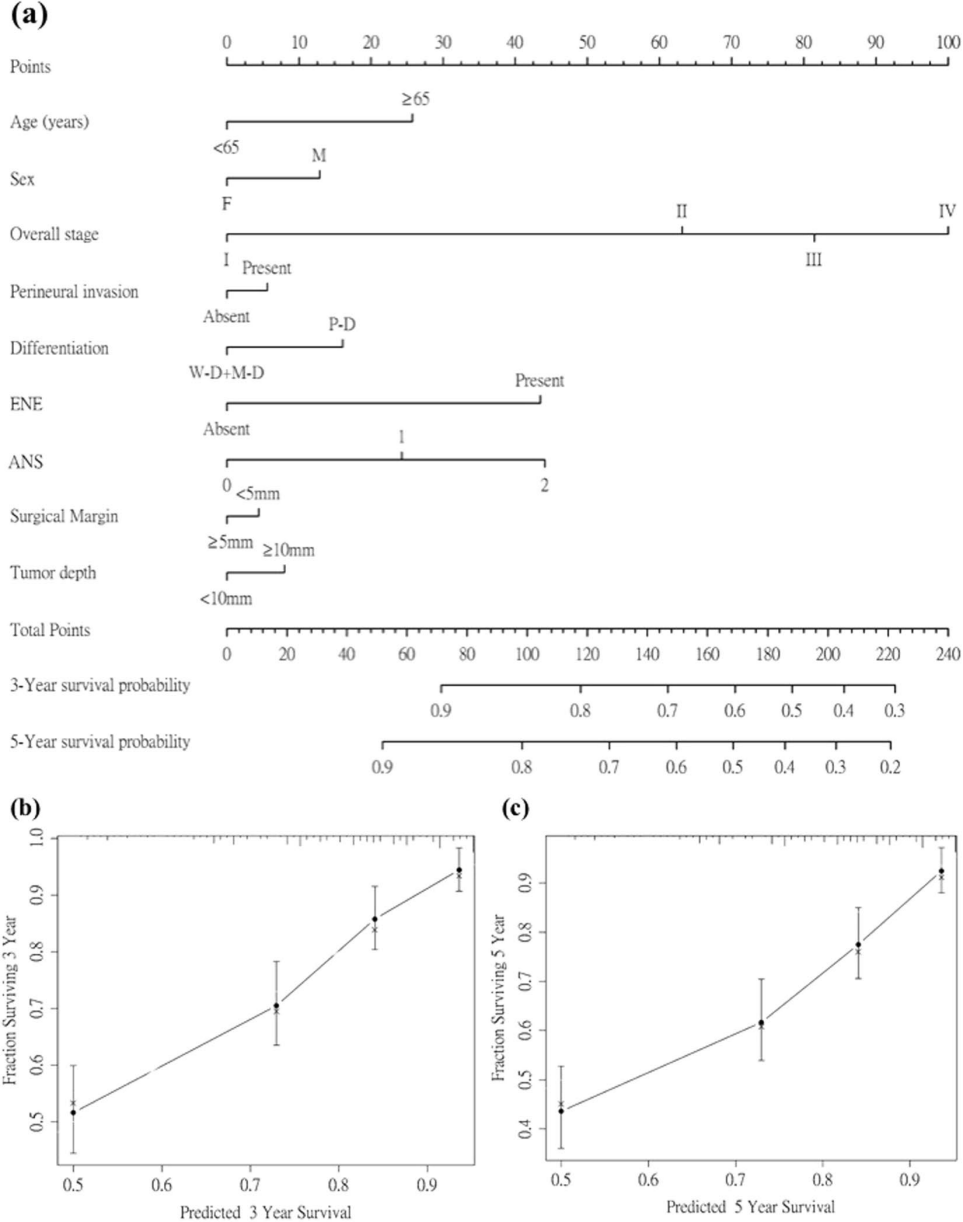
Nomogram and survival predictions. (**a**) Nomogram for OS prediction. A vertical line is drawn from each factor to the point score. By adding the points from all factors, a total points score is reached, which is translated into 3-year and 5-year OS probabilities by drawing a vertical line to its axis. Calibration plots of the nomogram to predict (**b**) 3-year OS and (**c**) 5-year OS. The blue line indicates the ideal prediction, and the black line represents the nomogram’s performance. Black dots with bars represent the nomogram’s performance with 95% CI when applied to the observed surviving cohorts. Abbreviations: ENE = extranodal extension; ANS = albumin/NLR score.

The nomogram for calculating the 3-year and 5-year OS is presented in Fig. 2 as well as the calibration plots for 3-year and 5-year OS probability. The 95% confidence intervals of the 3- and 5-year survival probablilities were [0.771, 0.841] and [0.702, 0.787], respectively. The c-index for the nomogram of tumor staging alone was 0.688, whereas the c-indexes of the nomograms that included all serum/ clinicopathological factors with and without ANS were 0.750 and 0.740, respectively.

## Discussion

Currently, TNM staging is the most common prognostic tool for determining OSCC prognosis. TNM staging is reliable but is a static tool that focuses only on tumor-specific characteristics without accounting for other factors. Other adjuncts to provide the surgeon, and more importantly, the patient, with better preoperative prognostic information would be useful. Nomograms are a pictorial representation of a complex mathematical formula that have emerged as a simpler, yet more advanced method compared to TNM staging. By integrating diverse prognostic and determinant variables to generate the probability of a clinical event, the nomogram fulfills a necessary role in oncological personalized medicine^24^.

In this study, we developed a nomogram that includes preoperative hematological and laboratory markers of systemic inflammatory responses to improve prognosis prediction in OSCC patients after surgery. A decreased serum albumin level and higher NLR before surgery were the independent and negative predictors of OS. A prognostic score, named as ANS, was created by combining serum albumin levels with NLR. An elevated score is a risk factor for long-term outcomes and is associated with high overall stage, ENE, perineural invasion, and tumor depth. According to our previous studies, ENE, cell differentiation, and perineural invasion have been demonstrated as important prognostic factors^25–27^. Consequently, the ANS, incorporated with TNM stage, age, sex, ENE, cell differentiation, and perineural invasion, was constructed as this prognostic nomogram to predict OS. Compared to the established serum/clinical model without ANS, the c-index of the model including ANS could increase from 0.740 to 0.750, showing an potential role of ANS on the prognostic prediction in the nomogram model.

Previous studies showed that several inflammatory biomarkers can be used as prognostic tools for different cancer types. Several blood cell-based prognostic biomarkers in relation to systemic inflammation responses were established to predict patient outcomes. These included NLR, lymphocyte-to-macrophage ratio (LMR), platelet-to-lymphocyte ratio (PLR), and hemoglobin level, which can each be used as a single prognostic indicator or can be combined with others. Several reports have demonstrated that a higher NLR and PLR, or a lower LMR correlates with a poor prognosis in head and neck cancer patients^22,28–30^. However, the cut-off values for these inflammatory biomarkers vary. The cut-off value identified by one single cohort may not apply to other independent cohorts. Although the interactions between the tumor cells and the host immune system have not been fully elucidated, inflammation clearly plays a pivotal role and has been recognized as a hallmark of cancer^31^. An elevated NLR indicates an imbalance between the innate and acquired immune response. Lymphocytes are a part of the more specific, acquired immune response and attack tumors by recognizing tumor antigens^32^. Tumors with higher infiltration by lymphocytes has been linked with better prognosis in several cancers^33,34^. Neutrophils, on the other hand, are a part of the innate, non-specific immune response and can secrete tumor growth promoting factors which may enable tumor growth and spread^35,36^. A high number of intratumoral neutrophils has been linked to poorer prognosis in renal cancer^37^. Thus, even though the exact pathways are not completely known, higher NLR-values indicate that the tumor has induced a stronger response in the innate immune response than in the acquired immune response, which might be linked to a poorer prognosis. To the best of our knowledge, this study evaluated the greatest amount of patient material for inflammation biomarkers and ratios with possible prognostic potential after OSCC surgery. The study showed that NLR, albumin and albumin/NLR scores have potential prognostic value for OS. Increased NLR was associated with decreased OS. The prognostic value mechanism for these inflammatory biomarkers in cancer remains unclear. Several reports have demonstrated that cancer and inflammation interact reciprocally^38^. An elevated NLR indicates an imbalance between the innate and acquired immune response, with increased cytokine levels such as interleukins^39,40^. This elevation may reflect an aggressive and/or advanced tumor as well as an inflammatory microenvironment that enables a tumor to spread; i.e., lymphocytes can have tumor suppressing effects, whereas neutrophils can create a favorable environment for the tumor by remodeling the extracellular matrix and angiogenesis, thus enabling the tumor to spread^41,42^.

Low serum albumin levels are reported to be associated with poor survival in several cancers. In this study, decreased serum albumin was also associated with decreased OS in OSCC patients. Albumin is a negative acute phase protein that decreases with inflammation and due to other reasons, such as malnutrition, increased age and disease^43–46^. The novel ANS has significant prognostic potential, as shown in the current nomogram. Creating a nomogram including this score, together with demographic and clinicopathological factors (age, tumor stage, perineural invasion, ENE and cell differentiation), contributed to the increased c-index of 0.750 compared to 0.688 using tumor stage alone to predict OS. The interactions between inflammation and cancer are intricate and unclear, but these findings emphasize their importance and provide a means for improving prognostic information through a rapid and economical blood test.

Nomograms have been used in several cancers as adjuncts in prognostic determination^8,47–49^. They can be tools to determine patient prognosis in addition to TNM classification, the current gold standard. The main advantage of the preoperative nomogram is its ability to estimate individualized 3-year and 5-year survival based on patient blood tests and disease characteristics. Nomograms can help surgeons identify patients who may derive greater benefit from more extensive surgery, thus affecting all aspects of cancer care. However, the present study has several limitations. First, the cut-off values for serum albumin and NLR were 4.5 and 2.28 based on this specific study population. When reviewing the literature, there are different cut-off values for biomarkers, ratios and scores based on cancer type and study population/area. Thus, it is difficult to create standardized cut-offs that can be used worldwide, even within a single cancer type^19,48,49^. Second, this dataset included only patients who underwent surgical resection for OSCC. Patients with unresectable tumors or who refused surgery were not enrolled. Third, this retrospective cohort consisted of OSCC patients over a 10-year follow-up period. A nomogram may become less accurate over time due to improved therapeutic strategies.

Although this study comprised a large population over a recent time period, it was a retrospective study with the weaknesses that this confers. Although internal validation was performed to prevent over-interpreting the data, external validation will verify if our findings are universally applicable. Additionally, a prospective study to confirm the results would be valuable, and we plan to pursue this in the near future.

## Conclusions

NLR and albumin have prognostic value for OS in OSCC after surgery. A nomogram was established by combining the ANS with demographic and clinicopathological variables. Regarding OS in preoperative decision making, this nomogram may provide more accurate prognostic information for patients and clinicians.

## Materials and Methods

### Patients and clinical specimens

This study retrospectively enrolled consecutive patients with OSCC tumors who were diagnosed at Chang Gung Memorial Hospital between September 2005 and December 2014. Patients with at least one of the following conditions were considered ineligible for study inclusion: other concomitant primary cancer (synchronous or metachronous), recurrent cancer, distant metastasis at presentation, prior history of malignancy, treatment with neoadjuvant therapy, or medical contraindication for surgery. A total of 613 patients were included. Patients were defined as betel nut chewers if they had chewed 2 or more betel nuts daily for at least 1 year; cigarette smokers if they smoked every day for at least 1 year; and alcohol drinkers if they consumed an alcoholic beverage 1 or more times per week for at least 6 months. All patients provided informed consent prior to study participation, and the study was approved by the Institutional Review Board of Chang Gung Memorial Hospital. Patients underwent standard preoperative work-ups per institutional guidelines, including a detailed medical history, complete physical examination, blood tests, computed tomography or magnetic resonance imaging scans of the head and neck, chest radiographs, bone scan, and abdominal ultrasound. Primary tumors were excised with margins per institutional guidelines, with intraoperative frozen section controls. Surgical defects were immediately reconstructed, if necessary, by plastic surgeons using local, pedicled or free flap. Following surgical treatment, the pathological TNM classification of all tumors was established per the American Joint Committee on Cancer Staging Manual (2010). Postoperative chemotherapy and/or radiotherapy was performed if indicated by our institutional guidelines. Briefly, Postoperative radiotherapy was performed on patients with pathologic T4 tumors and positive lymph nodes within 6 weeks following surgery. Patients with any pathologic findings such as metastasis in multiple neck lymph nodes, extracapsular spread, positive surgical margins, nodal dissemination at level 4 or 5, and perineural invasion received adjuvant concurrent chemoradiotherapy. The chemotherapy was a cisplatin-based regimen, and the total radiation dose was 66 Gy delivered in fractions of 2-Gy per day for 5 days per week. After discharge, all patients had regular follow-up visits every 2 months for the first year, every 3 months for the second year, and every 6 months thereafter.

NLR was calculated by dividing the absolute neutrophil count by the absolute lymphocyte count. ANS was calculated by assigning 0 or 1 point for albumin and for NLR levels above or below the cut-off value. This provided a possible score of 0–2. The cut-off was set as the median value.

### Statistical analysis and stratification

All statistical data are expressed as the mean ± SD. The chi-square and Wilcoxon tests were used to compare clinicopathological characteristics in OSCC tumors. All patients underwent follow-up evaluations at our outpatient clinic until December 2016 or death. Survival analyses were plotted using the Kaplan-Meier method, and differences were evaluated using the log-rank test. A Cox regression model was used to perform the univariate and multivariate analyses. Statistical analyses were performed using SAS software (version 9.1; SAS Institute, Cary, NC). All *p*-values were two-sided, and statistical significance was set at *p* < 0.05. The nomogram was created using the R software “rms” package (Version 5.1–0, Vanderbilt University, Nashville, TN). The concordance index (c-index) was calculated to determine the nomogram’s ability to predict OS. A value of 0.5 indicates random predictability and 1.0 represents perfect predictability^50^. For example, in randomly selected patient pairs, a calculated value of 0.6 indicated a 60% probability that patients who were predicted to have poor OS would have shorter OS. The c-index was calculated for the current standard of OS prediction (TNM classification) as well as for the proposed nomogram.

## References

[1] Ferlay J (2015). Cancer incidence and mortality worldwide: sources, methods and major patterns in GLOBOCAN 2012. Int J Cancer.

[2] Chinn SB, Myers JN (2015). Oral Cavity Carcinoma: Current Management, Controversies, and Future Directions. J Clin Oncol.

[3] Po Wing Yuen A (2002). Prognostic factors of clinically stage I and II oral tongue carcinoma-A comparative study of stage, thickness, shape, growth pattern, invasive front malignancy grading, Martinez-Gimeno score, and pathologic features. Head Neck.

[4] O-charoenrat P (2003). Tumour thickness predicts cervical nodal metastases and survival in early oral tongue cancer. Oral Oncol.

[5] Paleri V (2010). Comorbidity in head and neck cancer: a critical appraisal and recommendations for practice. Oral Oncol.

[6] Hsu WL, Yu K, Chiang CJ, Chen TS, Wang CP (2017). Head and neck cancer incidence trends in Taiwan, 1980~2014. Int J Head Neck Science.

[7] Sobin LH (2003). TNM: evolution and relation to other prognostic factors. Semin Surg Oncol.

[8] Montero PH (2014). Nomograms for preoperative prediction of prognosis in patients with oral cavity squamous cell carcinoma. Cancer.

[9] Mantovani A (2005). Cancer: inflammation by remote control. Nature.

[10] Coussens LM, Werb Z (2002). Inflammation and cancer. Nature.

[11] Jakobisiak M, Lasek W, Golab J (2003). Natural mechanisms protecting against cancer. Immunol Lett.

[12] Orange JS, Ballas ZK (2006). Natural killer cells in human health and disease. Clin Immunol.

[13] Shrotriya S, Walsh D, Bennani-Baiti N, Thomas S, Lorton C (2015). C-Reactive Protein Is an Important Biomarker for Prognosis Tumor Recurrence and Treatment Response in Adult Solid Tumors: A Systematic Review. PloS one.

[14] Gupta D, Lis CG (2010). Pretreatment serum albumin as a predictor of cancer survival: a systematic review of the epidemiological literature. Nutrition journal.

[15] Templeton AJ (2014). Prognostic role of neutrophil-to-lymphocyte ratio in solid tumors: a systematic review and meta-analysis. J Natl Cancer Inst.

[16] Zhao QT (2016). Prognostic role of platelet to lymphocyte ratio in non-small cell lung cancers: A meta-analysis including 3,720 patients. Int J Cancer.

[17] Hutterer GC (2014). Low preoperative lymphocyte-monocyte ratio (LMR) represents a potentially poor prognostic factor in nonmetastatic clear cell renal cell carcinoma. Urol Oncol.

[18] Karakiewicz PI (2007). Platelet count and preoperative haemoglobin do not significantly increase the performance of established predictors of renal cell carcinoma-specific mortality. European urology.

[19] Chang Y (2015). Systemic inflammation score predicts postoperative prognosis of patients with clear-cell renal cell carcinoma. Br J Cancer.

[20] Forrest LM, McMillan DC, McArdle CS, Angerson WJ, Dunlop DJ (2003). Evaluation of cumulative prognostic scores based on the systemic inflammatory response in patients with inoperable non-small-cell lung cancer. Br J Cancer.

[21] Perisanidis C (2013). High neutrophil-to-lymphocyte ratio is an independent marker of poor disease-specific survival in patients with oral cancer. Medical oncology (Northwood, London, England).

[22] Chen S (2016). The preoperative platelet-lymphocyte ratio versus neutrophil-lymphocyte ratio: which is better as a prognostic factor in oral squamous cell carcinoma?. Therapeutic advances in medical oncology.

[23] Kattan MW (2002). Nomograms. Introduction. Semin Urol Oncol.

[24] Grimes DA (2008). The nomogram epidemic: resurgence of a medical relic. Annals of internal medicine.

[25] Fang KH (2009). Histological differentiation of primary oral squamous cell carcinomas in an area of betel quid chewing prevalence. Otolaryngol Head Neck Surg.

[26] Adel M (2015). Evaluation of Lymphatic and Vascular Invasion in Relation to Clinicopathological Factors and Treatment Outcome in Oral Cavity Squamous Cell Carcinoma. Medicine.

[27] De Paz D, Kao HK, Huang Y, Chang KP (2017). Prognostic Stratification of Patients With Advanced Oral Cavity Squamous Cell Carcinoma. Curr Oncol Rep.

[28] He JR (2012). Pretreatment levels of peripheral neutrophils and lymphocytes as independent prognostic factors in patients with nasopharyngeal carcinoma. Head Neck.

[29] Tu XP (2015). Preoperative neutrophil-to-lymphocyte ratio is an independent prognostic marker in patients with laryngeal squamous cell carcinoma. BMC cancer.

[30] Rachidi S (2016). Neutrophil-to-lymphocyte ratio and overall survival in all sites of head and neck squamous cell carcinoma. Head Neck.

[31] Hanahan D, Weinberg RA (2011). Hallmarks of cancer: the next generation. Cell.

[32] Boon T, Coulie PG, Van den Eynde B (1997). Tumor antigens recognized by T cells. Immunol Today.

[33] Galon J (2006). Type, density, and location of immune cells within human colorectal tumors predict clinical outcome. Science.

[34] Loi S (2013). Prognostic and predictive value of tumor-infiltrating lymphocytes in a phase III randomized adjuvant breast cancer trial in node-positive breast cancer comparing the addition of docetaxel to doxorubicin with doxorubicin-based chemotherapy: BIG 02-98. J Clin Oncol.

[35] Jabłońska E (2001). TNF-alpha, IL-6 and their soluble receptor serum levels and secretion by neutrophils in cancer patients. Arch Immunol Ther Exp (Warsz).

[36] McCourt M, Wang JH, Sookhai S, Redmond HP (1999). Proinflammatory mediators stimulate neutrophil-directed angiogenesis. Arch Surg.

[37] Jensen HK (2009). Presence of intratumoral neutrophils is an independent prognostic factor in localized renal cell carcinoma. J Clin Oncol.

[38] Grivennikov SI, Greten FR, Karin M (2010). Immunity, inflammation, and cancer. Cell.

[39] Kantola T (2012). Stage-dependent alterations of the serum cytokine pattern in colorectal carcinoma. Br J Cancer.

[40] Motomura T (2013). Neutrophil-lymphocyte ratio reflects hepatocellular carcinoma recurrence after liver transplantation via inflammatory microenvironment. J Hepatol.

[41] Mantovani A, Allavena P, Sica A, Balkwill F (2008). Cancer-related inflammation. Nature.

[42] Dumitru CA, Lang S, Brandau S (2013). Modulation of neutrophil granulocytes in the tumor microenvironment: mechanisms and consequences for tumor progression. Semin Cancer Biol.

[43] Don BR, Kaysen G (2004). Serum albumin: relationship to inflammation and nutrition. Semin Dial.

[44] Salive ME (1992). Serum albumin in older persons: relationship with age and health status. J Clin Epidemiol.

[45] Campion EW, deLabry LO, Glynn RJ (1988). The effect of age on serum albumin in healthy males: report from the Normative Aging Study. J Gerontol.

[46] Liu SA (2006). Nutritional factors and survival of patients with oral cancer. Head Neck.

[47] Kattan MW, Eastham JA, Stapleton AM, Wheeler TM, Scardino PT (1998). A preoperative nomogram for disease recurrence following radical prostatectomy for prostate cancer. J Natl Cancer Inst.

[48] Li Y (2016). Nomograms for predicting prognostic value of inflammatory biomarkers in colorectal cancer patients after radical resection. Int J Cancer.

[49] Guthrie GJ (2013). The systemic inflammation-based neutrophil-lymphocyte ratio: experience in patients with cancer. Crit Rev Oncol Hematol.

[50] Harrell, F. E. Jr., Lee, K. L. & Mark, D. B. Multivariable prognostic models: issues in developing models, evaluating assumptions and adequacy, and measuring and reducing errors. *Stat Med* **15**, 361–387, doi:10.1002/(SICI)1097-0258(19960229)15:4<361::AID-SIM168>3.0.CO;2-4 (1996). 10.1002/(SICI)1097-0258(19960229)15:4<361::AID-SIM168>3.0.CO;2-4 8668867

